# Reviewed article: Pires MCS, Silva MJS, Araújo PP, Retto MPF. Use of medications in women with triple-negative breast cancer between 2018 and 2019 in a Brazilian public hospital: a retrospective study. Epidemiol Serv Saude. 2025:34;e20240180. 10.1590/S2237-96222024v33e20240180.en

**DOI:** 10.1590/S2237-96222025v34e20240180.a

**Published:** 2025-02-21

**Authors:** Anke Bergmann

**Affiliations:** 1 Instituto Nacional de Câncer: Rio de Janeiro, RJ, Brazil

## Peer review A

## Questionnaire

Does the manuscript contain new and significant information to justify publication? Yes

Does the Abstract (Summary) clearly and accurately describe the content of the article? Yes

Is the problem significant and concisely stated? Yes

Are the methods described comprehensively? Yes

Are the interpretations and conclusions justified by the results? No

Is adequate reference made to other work in the field? Yes

## Manuscript Structure

Length of article is: Adequate

Number of tables is: Adequate

Number of figures is: Adequate

Please state any conflict(s) of interest that you have in relation to the review of this paper (state “none” if this is not applicable).

None

Interest: Good

Quality: Good

Originality: Good

Overall: Good

Recommendation: Minor Revision

## Comments

Trata-se de um estudo descritivo unicentrico com 176 mulheres com câncer de mama triplo negativo, com o objetivo de caracterizar o perfil de utilização de medicamentos com finalidade inicial neoadjuvante e adjuvante, com o intuito de compreender o padrão de tratamento adotado. Algumas considerações: 

Resumo: A conclusão apresentada no resumo não pode ser obtida com o estudo descritivo. Sugiro rever a afirmação.

Introdução

Está bem descrita, com citações atuais e relevantes.

Métodos

Sugiro incluir o cálculo do tamanho amostral ou análise post hoc.

Resultados

Sugiro incluir percentual após a citação do “n” em todo o resultado “Durante o período de tratamento sistêmico, registrou-se o óbito de 58 pacientes...”; Na tabela 1, colocar o n no titulo e incluir a categoria” ausência de informações”; Na figura 1, incluir os percentuais após o número absoluto.

Discussão

Inserir as conclusões ao final da discussão.

